# Surgical Management and Reconstruction of Hoffman's Disease (Dissecting Cellulitis of the Scalp)

**DOI:** 10.1155/2016/2123037

**Published:** 2016-02-07

**Authors:** Justin M. Hintze, Brittany E. Howard, Carrlene B. Donald, Richard E. Hayden

**Affiliations:** ^1^Trinity College, University of Dublin, College Green, Dublin 2, Ireland; ^2^Department of Otolaryngology-Head and Neck Surgery, Mayo Clinic Arizona, 5777 E. Mayo Boulevard, Phoenix, AZ 85054, USA

## Abstract

Dissecting cellulitis of the scalp, or Hoffman's disease, is a rare dermatologic condition characterized by recurrent pustules and sinus tract formation leading to scarring and alopecia. Medical management includes the use of corticosteroids, antibiotics, isotretinoin, and adalimumab. In cases where the disease is severe, refractory, and intractable, surgery is an option. We report two cases of Hoffman's disease, where medical management failed to achieve remission. Surgical treatment was undertaken with complete resection of the affected scalp in staged procedures with subsequent split-thickness skin grafting for reconstruction. Surgery achieved both disease remission and excellent aesthetic outcomes in both patients.

## 1. Introduction

Dissecting cellulitis of the scalp (Hoffman's disease or perifolliculitis capitis abscedens et suffodiens) is a chronic inflammatory condition characterized by recurrent suppurative and tender pustules and sinus tract formation and often leading to scarring and alopecia most commonly affecting the hair bearing regions of the scalp [1]. It is part of the “follicular occlusion triad,” along with acne conglobate and hidradenitis suppurativa [2], and occurs most frequently in adult African American men between the second and fourth decades of life [1]. It may be associated with sternoclavicular hyperkeratosis, polyarticular arthritis, or human leukocyte antigen-B27 negative spondyloarthropathies [3]. Traditional management has focused on medical treatments and interventions. We present our experience with surgical management of refractory and severe disease.

## 2. Case Report

Patient 1 was a 30-year-old Hispanic male who presented to the Mayo Clinic with multiple abscesses and sinus tracts of the scalp associated with intractable pain and was subsequently diagnosed with dissecting cellulitis of the scalp based on pathological findings from a punch biopsy. Pathology showed destruction of follicular units with extensive perifollicular inflammatory response with granulomas, abscesses, and sinus tracts. He was initially managed with a combination of sulfamethoxazole/trimethoprim, cephalexin, and ciprofloxacin. Steroids were avoided due to a history of diabetes mellitus. In addition, he was prescribed dapsone and isotretinoin and also received Kenalog injections for local control of the scar tissue. Following the failure of these medical interventions, a staged resection of the diseased scalp was recommended. To achieve complete resection of the disease, surgical resection was performed just superficial to the galeal level as 3 staged procedures. As his disease involved all hair bearing scalp, this was removed in its entirety. This was not performed as a single stage procedure due to the extent of surgery that would be required, need for intraoperative repositioning, and associated surgical time requirements. Postoperatively after each resection, wet-to-dry dressings were applied to encourage a granulation base and reconstruction with a partial-thickness skin graft was performed as a second procedure once an appropriate granulation tissue base had developed. At 1-year follow-up, the patient remained disease-free.

**Figure 1:**
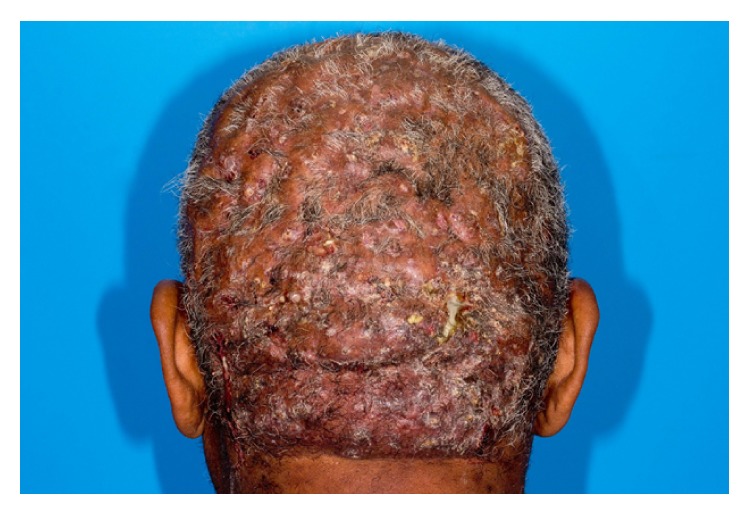
Preoperative appearance of the patient's diseased scalp showing multiple discharging pustules, diffuse alopecia, and fibrosis.

**Figure 2:**
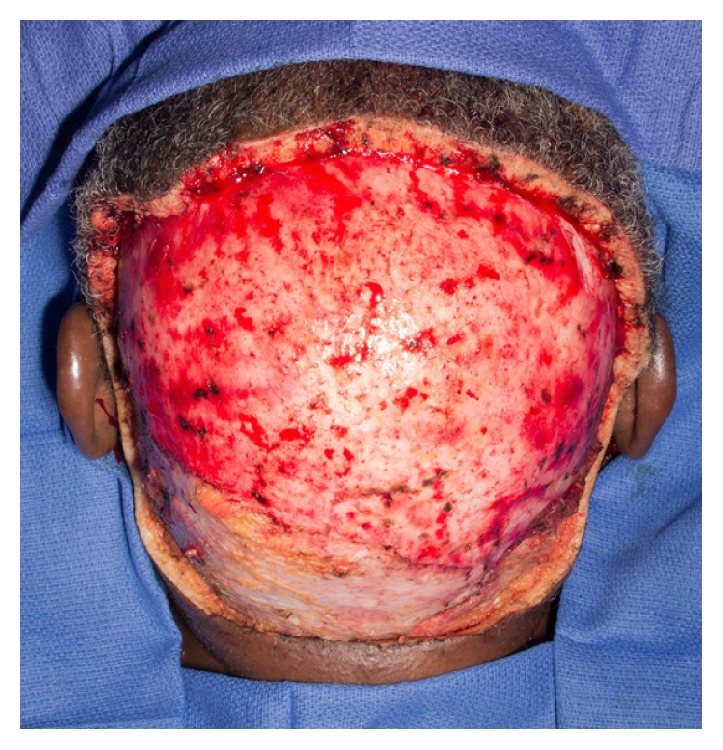
Intraoperative image showing removed diseased scalp to a level just below the galea with preservation of the periosteum.

**Figure 3:**
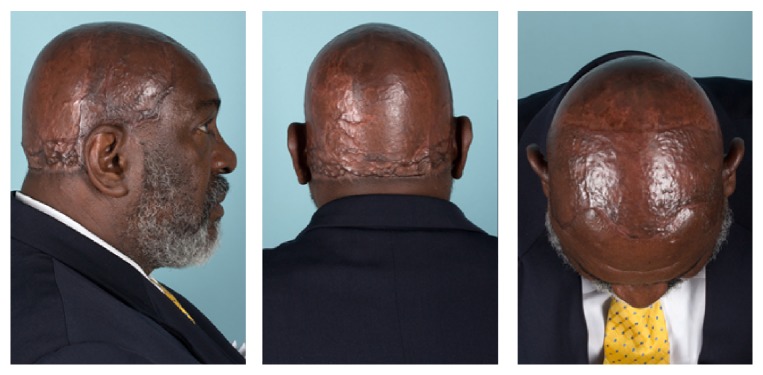
Postoperative (1 year) appearance showing good take and healing of the split-thickness skin graft.

Patient 2, a 58-year-old African American male, was referred to the otorhinolaryngology service from the dermatology department for evaluation of extensive dissecting cellulitis of the scalp and posterior neck. Pathological diagnosis was confirmed with punch biopsy with typical histopathological findings including extensive perifollicular inflammation with neutrophil infiltrate, abscess cavities, and sinus tracts. His disease had been progressive across the prior decade and was refractory to medical treatment including multiple trials of clindamycin, rifampicin, metronidazole, dapsone, isotretinoin, and adalimumab. He showed extensive disease involving the entirety of the hair bearing scalp with pustules, sinus tracts, scarring, and patchy alopecia (Figure 1). He was determined to be a surgical candidate given his lack of response to medical management. Due to the scale of the disease and need to remove all hair bearing scalp to clear his disease, excision was planned as staged excision procedures with reconstruction following each resection. This eliminated the need for repositioning intraoperatively and shortened the procedure times and extent of donor site injury at each stage. The patient's disease extended beyond the level of the galea, requiring a subgaleal resection to fully eradicate the disease (Figure 2). The first stage excision of the posterior scalp was followed by wet-to-dry dressing treatment of the resection site to encourage granulation tissue formation. Once appropriate granulation base had developed, a split-thickness skin graft from the anterolateral thigh was used for reconstruction. A vacuum-assisted closure dressing was used to encourage graft take and removed 10 days postoperatively. An identical staged resection and reconstruction of the anterior scalp were then performed. At one-year follow-up, the patient remains disease-free and shows excellent aesthetic outcome (Figure 3).

## 3. Literature Review

Original descriptions of the disease are credited to Spitzer [4] in 1903, but it was Barney [5] in 1931 who first coined the terminology dissecting cellulitis of the scalp to describe the disease. Dissecting cellulitis is part of the follicular occlusion triad associated with hidradenitis suppurativa and acne conglobate. Although these diseases have similar pathogenesis and often can be concomitant, they are distinct disease entities. The pathogenesis of dissecting cellulitis of the scalp involves follicular hyperkeratosis causing occlusion of the pilosebaceous apparatus. Subsequent follicular dilation and potential secondary infection can occur. This in turn can lead to follicular rupture, perifolliculitis, and a neutrophilic and granulomatous inflammatory response [1]. As a result of these repeated episodes of inflammation and infection abscesses and sinus tracts form with eventual scarring and alopecia [6]. The most concerning end points of disease include an increased risk for osteomyelitis of the underlying calvarium and squamous cell carcinoma within the diseased cutaneous tissue [3, 7]. Histopathologically, dissecting cellulitis is characterized by acneiform distension of the follicles with perifollicular inflammation early in the disease process.

There is no consensus regarding the optimal medical management of dissecting cellulitis of the scalp as the rarity of the disease results in data being limited to case reports and small case series. Initial treatments typically include any combination of improved scalp hygiene, antiseptic, topical antibiotics, lesional aspiration, oral antibiotics, and corticosteroid injections for mild disease [3]. Alternatively, isotretinoin can be considered as a first-line therapy [7]. In more severe cases of the disease, systemic antibiotics (such as rifampicin) and steroids are traditionally used with mixed results of efficacy. Most recently, there has been interest in the possible role of monoclonal antibodies that can play in the treatment of the underlying inflammatory pathway of the disease. In a case series of 3 patients with dissecting cellulitis of the scalp and treated with adalimumab, clinical remission was achieved in all 3 cases after 8 weeks with some evidence of residual pathological changes. However, when treatment was terminated, the patient's symptoms returned within 4 weeks [8].

For severe, intractable, and refractory cases of dissecting cellulitis of the scalp, surgical management is an option. Our experience supports prior reports of disease eradication with partial thickness scalp resection [1, 3, 9, 10]. Scalp resection is performed to a level just deep to the disease, usually galeal or just subgaleal. It is crucial that at least the periosteum is preserved so that there is a base upon which reconstruction can be performed. Excellent aesthetic outcomes can then be achieved with a reconstruction by split-thickness skin grafting. We prefer to perform this in a separate procedure after cultivating a base of granulation tissue using a combination of wet-to-wet dressing followed by vacuum-assisted closure (VAC) dressing to increase likelihood of complete skin graft survival. In our experience, surgical treatment has resulted in complete resolution of disease. There is only one case report of disease recurrence following surgical intervention [11].

Severe cases of dissecting cellulitis of the scalp can lead to marked disfigurement, poor cosmetic appearance, and bad odor [3]. This may cause significant psychological distress and loss of quality of life. Prior reports and our own experience support that patients experience significantly improved quality of life following surgical resection and reconstruction of recalcitrant disease [10]. As such, in this subset of patients, early surgical intervention can be considered.

## 4. Conclusion

Dissecting cellulitis of the scalp is a rare condition mostly affecting men aged 20 to 40. It is characterized by recurrent suppurative and tender pustules and sinus tract formation that may advance to scarring and alopecia. Medical management includes the use of antibiotics, corticosteroids, isotretinoin, and antitumor necrosis factor medication. Surgical excision is an option for cases refractory to medical therapy and severe disease. This involves scalp resection to a level deep to the disease, followed by split-thickness skin grafting. There may be significant psychosocial comorbidities associated with this disfiguring condition, so earlier resection may be considered. Further research is required to establish role of early surgical intervention and to monitor long-term surgical outcome.
